# PARP2 poly(ADP-ribosyl)ates nuclear factor erythroid 2-related factor 2 (NRF2) affecting NRF2 subcellular localization

**DOI:** 10.1038/s41598-023-35076-w

**Published:** 2023-05-15

**Authors:** Laura Jankó, Emese Tóth, Miklós Laczik, Boglárka Rauch, Eszter Janka, Bálint L. Bálint, Péter Bai

**Affiliations:** 1 Department of Medical Chemistry, Faculty of Medicine, University of Debrecen, Egyetem Tér 1., 4032 Debrecen, Hungary; 2 Center of Excellence, The Hungarian Academy of Sciences, Budapest, Hungary; 3 Department of Biochemistry and Molecular Biology, Faculty of Medicine, University of Debrecen, Debrecen, 4032 Hungary; 4 Department of Dermatology, Faculty of Medicine, University of Debrecen, Debrecen, 4032 Hungary; 5 Department of Bioinformatics, Semmelweis University, Tűzoltó Utca 7–9., Budapest, 1094 Hungary; 6 MTA-DE Lendület Laboratory of Cellular Metabolism, Debrecen, 4032 Hungary; 7 Research Center for Molecular Medicine, Faculty of Medicine, University of Debrecen, Debrecen, 4032 Hungary; 8 MTA-DE Cell Biology and Signaling Research Group ELKH, Debrecen, Hungary

**Keywords:** Cell signalling, Nuclear transport, Post-translational modifications, Transcription, Biochemistry

## Abstract

PARP2 is a member of the PARP enzyme family. Although, PARP2 plays role in DNA repair, it has regulatory roles in mitochondrial and lipid metabolism, it has pivotal role in bringing about the adverse effects of pharmacological PARP inhibitors. Previously, we showed that the ablation of PARP2 induces oxidative stress and, consequently, mitochondrial fragmentation. In attempt to identify the source of the reactive species we assessed the possible role of a central regulator of cellular antioxidant defense, nuclear factor erythroid 2-related factor 2 (NRF2). The silencing of PARP2 did not alter either the mRNA or the protein expression of NRF2, but changed its subcellular localization, decreasing the proportion of nuclear, active fraction of NRF2. Pharmacological inhibition of PARP2 partially restored the normal localization pattern of NRF2 and in line with that, we showed that NRF2 is PARylated that is absent in the cells in which PARP2 was silenced. Apparently, the PARylation of NRF2 by PARP2 has pivotal role in regulating the subcellular (nuclear) localization of NRF2. The silencing of PARP2 rearranged the expression of genes encoding proteins with antioxidant function, among these a subset of NRF2-dependent genes.

## Introduction

Cellular redox control and response to oxidative damage is vital for cell survival^1–3^. An intricate and widespread web of proteins are involved in conferring protection to cells against oxidative stress^1–3^. PARP1, PARP2 and PARP3 enzymes that belong to the PARP enzyme family, can readily respond to oxidative-stress induced DNA strand breaks and induce different forms of cell death^4–7^. The majority of cellular PARP activity can be attributed to PARP1 and PARP2, of which PARP1 is responsible for the majority^8–10^. PARP1 and PARP2 can introduce poly(ADP-ribose) (PAR) chains onto target proteins^10–17^ that can subsequently change their biological activity by modifying the biochemical activity or cellular localization^18^. PARP1 and PARP2 share multiple functions, encompassing their involvement in DNA repair^19,20^ or transcriptional regulation^19,21^. PARP2 is related to oxidative stress-associated pathologies and has pathological roles in these scenarios^8,22–26^. We recently showed that the silencing of PARP2 induces oxidative, but not nitrosative stress in different cellular models^27^. The source of the reactive oxygen species (ROS) is partly mitochondrial, however there are still unidentified sources^27^. We set out to identify the source of the remainder of reactive species in PARP2-silenced cells.

Multiple PARP inhibitors were developed for the pharmacological modulation of cellular PARP activity^28–30^. These inhibitors inhibit multiple PARP enzymes^31^. A subset of these inhibitors is now available in the clinical practice^28–30^ of which olaparib (Lynparza^®^) was the first to be registered^32,33^ Olaparib is an anticancer drug registered for the treatment of ovarian cancer, nevertheless, clinical studies are carried out to encompass the treatment of breast, prostate and pancreatic cancer^28–30^. Although in ovarian, breast and pancreatic cancer the use of olaparib is limited to the patients carrying BRCA mutations, it appears that the deficiency of other DNA repair factors may also sensitize cells for pharmacological PARP inhibition.

Oxidative stress may be elicited by a decrease in the activity or expression of antioxidant defense systems. Nuclear factor erythroid 2 (NFE2)-related factor 2 (Nfe2l2, or NRF2) is a transcription factor that has predominant role in regulating the expression of a set of antioxidant genes^34–36^. NRF2 forms a complex with the Kelch Like-ECH-Associated Protein 1 (KEAP1) that co-localizes NRF2 to Cul-3-RBx1-E3 (Cullin-3-ring box-1 E3) ubiquitin ligase complex for degradation^37–40^. However, KEAP1 has several redox-sensitive cysteine residues that are oxidized upon oxidative stress^41,42^ and, hence, KEAP1 releases NRF2, preventing its degradation^43–46^. NRF2 then translocates to the nucleus and dimerizes with other transcription factors, for example the members of the Maf family^47,48^. The heterodimers bind to EpRE/ARE-response elements in gene promoters and drive their transcription^47,49^. There are over 200 NRF2-dependent genes in humans, the majority of which are enzymes that detoxify cellular oxidative damage^35,50–52^.

PARP1 was shown to regulate NRF2-dependent transcription through binding to Maf proteins and, through that, modulate the DNA-binding capacity of NRF2^53,54^. Furthermore, there are important overlaps in the function of PARP enzymes and NRF2 in multiple organ systems, for example in the cardiovascular system^3,52,55–57^. Given the structural and functional overlap between PARP1 and PARP2^19,20^ we set out to assess whether there are connections between NRF2 and PARP2 and whether the deregulation of NRF2 could be the source of oxidative stress upon PARP2 silencing.

## Results

### PARP2 PARylates NRF2 and regulates its intracellular localization

**Figure 1:**
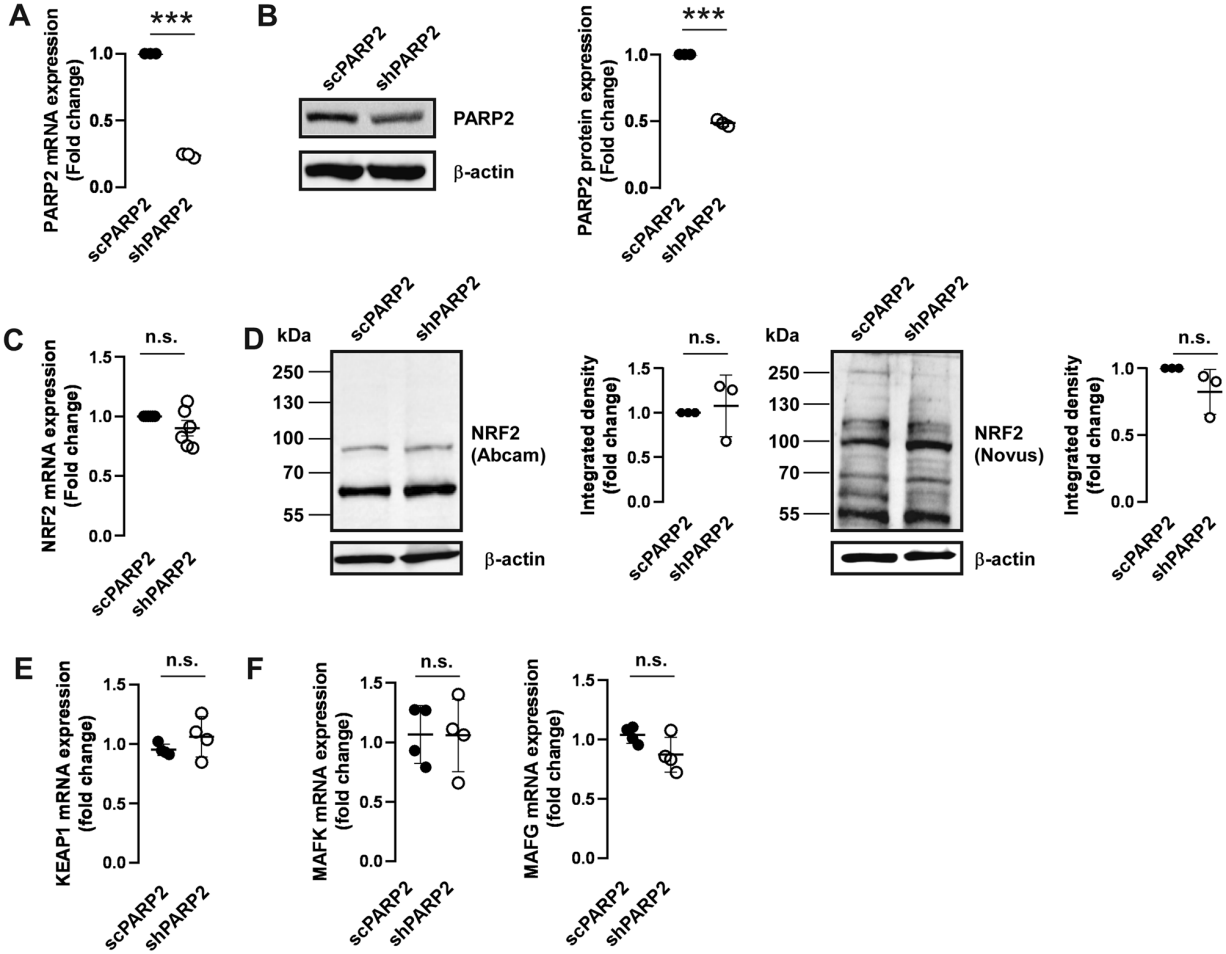
The silecing of PARP2 does not interfere with the expression of the elements of the NRF2 system. (**A**,**B**) 200 000 cells from scPARP2 and shPARP2 C2C12 cells were seeded into a 6-well plate and the expression of PARP2 was determined by (**A**) RT-qPCR (n = 3) and (**B**) Western blotting (n = 3). (**C**,**D**) 200 000 cells from scPARP2 and shPARP2 C2C12 cells were seeded into a 6-well plate and the expression of NRF2 was determined by (**C**) RT-qPCR (n = 6) and (**D**) Western blotting (n = 3) using the antibodies indicated. **(E**,**F)** 200 000 cells from scPARP2 and shPARP2 C2C12 cells were seeded into a 6-well plate and the expression of (**E**) KEAP1 and (**F**) the indicated MAF genes was determined by RT-qPCR (n = 3). Numerical values are presented as the average ± SD. Statistical significance was determined using paired, two-tailed Student’s t-test. ***Represent statistically significant differences between the scPARP2 and shPARP2 C2C12 cells at p < 0.001. n.s.—not significant.

PARP2 has important metabolic roles and modulates mitochondrial metabolism in multiple metabolically active tissues^19,20,58,59^, among these, skeletal muscle^23,60,61^, therefore, we chose C2C12 cells as cellular model for our studies^23^. To assess changes to the function of NRF2 upon PARP2 silencing, we applied a C2C12 cell line pair in which PARP2 was downregulated using an shRNA approach (shPARP2 C2C12) and its isogenic counterpartner expressing a sequence that has no target in murine or human cells (scPARP2 C2C12)^23,62^. We verified the downregulation of PARP2 mRNA and protein expression in the shPARP2 C2C12 cell line as compared to the scPARP2 C2C12 cell line (Fig. 1A,B). Next, we showed that the silencing of PARP2 does not affect the mRNA expression of NRF2 (Fig. 1C). We also assessed the expression of the NRF2 protein using two different antibodies from different sources, as in the literature there is an ambiguity in the apparent molecular mass of NRF2 (68–130 kDa) and different antibodies detect different NRF2 bands^63–65^. We applied antibodies that were validated in a previous study^65^. Silencing of PARP2 did not change the protein expression of NRF2 significantly (Fig. 1D). Finally, we showed that the mRNA expression of other members of the NRF2 system (KEAP1, MAFG, MAFK) is not different between scPARP and shPARP2 C2C12 cells (Fig. 1E,F). We were unable to detect the mRNA transcript of MAFF.

**Figure 2:**
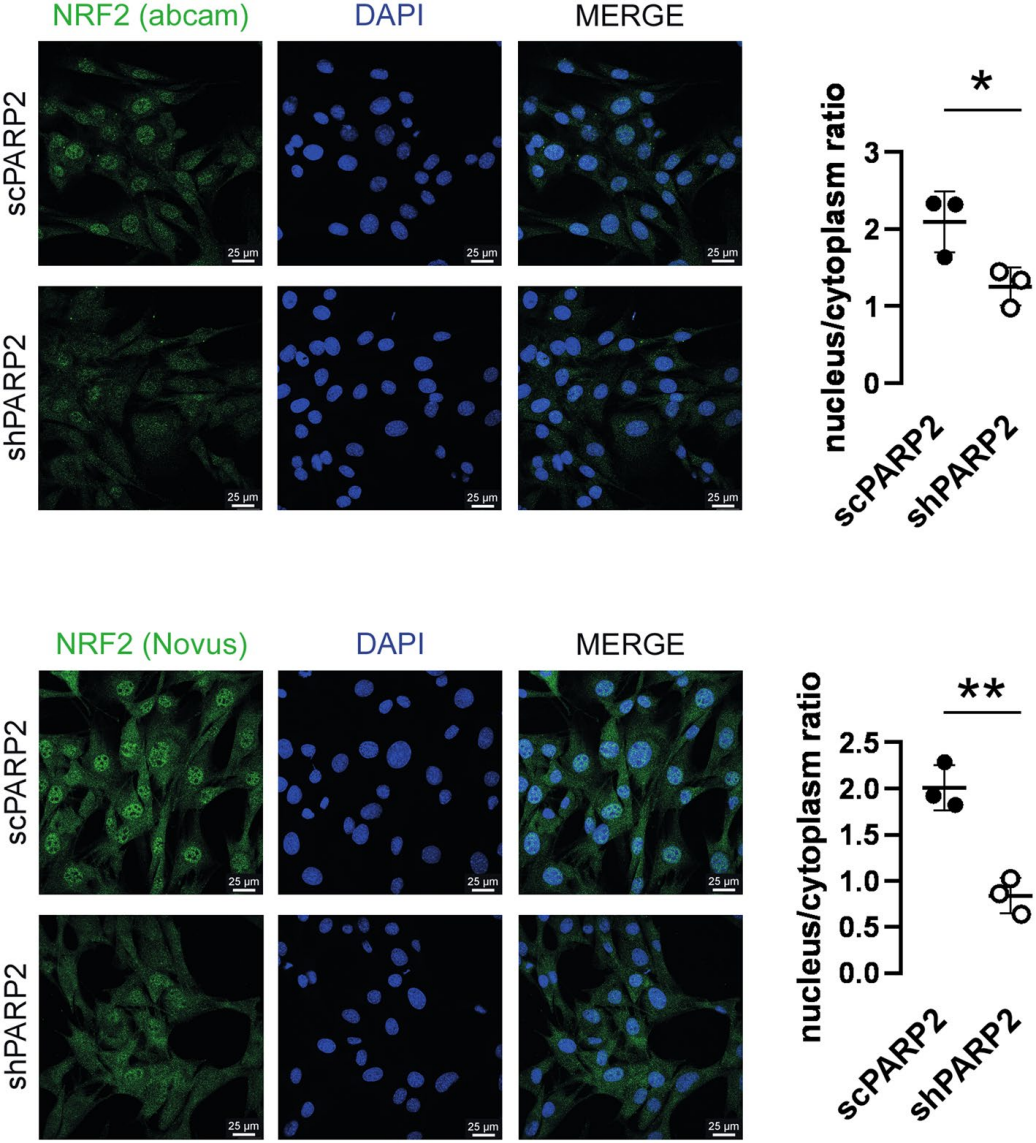
Silencing of PARP2 reduces the nuclear fraction of NRF2. 70 000 cells from scPARP2 and shPARP2 C2C12 cells were seeded into a 24-well plate on glass coverslips. The cells were stained with NRF2 specific antibody and nuclei were visualized using DAPI (n = 3). Images were analyzed using ImageJ software (50/50 cells were measured). For each biological replicate an average was generated that is depicted on the graph. Representative immunofluorescence images are presented in the figure. Numerical values are presented as the average ± SD. Statistical significance was determined using paired, two-tailed Student’s t-test. *Or **statistically significant differences between the scPARP2 and shPARP2 C2C12 cells at p < 0.05 or p < 0.01, respectively.

**Figure 3:**
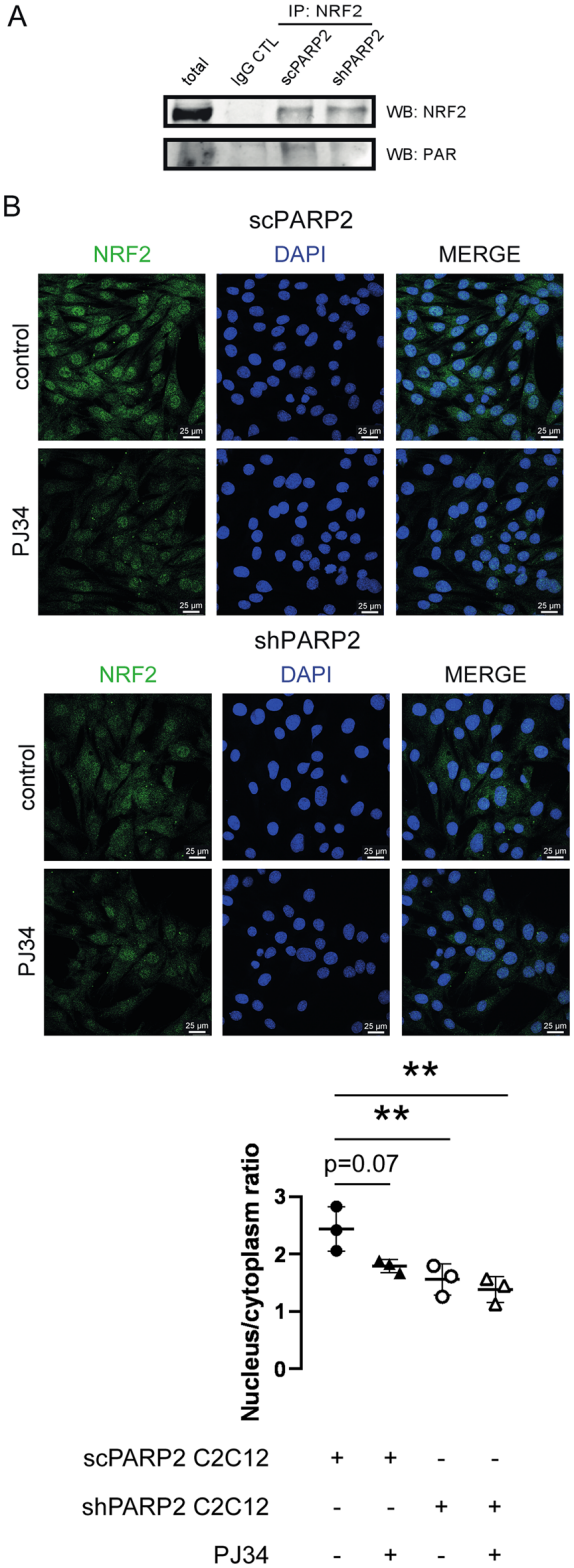
The PARylation of NRF2 by PARP2 is responsible for the nuclear localization of NRF2. (**A**) 200 000 cells from scPARP2 and shPARP2 C2C12 cells were seeded into a 6-well plate and PARylation level of NRF2 was determined by immunoprecipitating NRF2 and embranes were probed with NRF2 or PAR antibody in Western blotting (n = 2). (**B**) 40 000 cells from scPARP2 and shPARP2 C2C12 cells were seeded into a 24-well plate on glass coverslips and treated with 3 µM PJ34 for 24 h. After treatment, the cells were stained with NRF2 specific antibody and nuclei were visualized using DAPI (n = 3). Images were analyzed using ImageJ software (measured cells: 50 from each group). Representative Western blot images are presented in (**A**) and representative immunofluorescence images are presented in (**B**). Numerical values are presented as the average ± SD. Statistical significance was determined using ANOVA followed by Tukey’s post-hoc test. **Statistically significant differences between the scPARP2 and shPARP2 C2C12 cells at p < 0.01.

Next, we assessed the localization of NRF2. Silencing of PARP2 drastically decreased the nuclear localization of NRF2 (Fig. 2) suggesting that PARP2 is needed for the nuclear translocation of NRF2. PARP enzymes, among them PARP2, can produce large, branched and negatively charged polymers on target proteins^10^ that can modulate their localization and biochemical activity^66^. We hypothesized that PARP2 can PARylate NRF2. Indeed, when NRF2 was immunoprecipitated from cellular extracts we were able to detect PAR chains on the large molecular weight form of NRF2 protein (> 130 kDa; Fig. 3A). PAR chains were largely reduced in shPARP2 cells (Fig. 3A) suggesting a dominant role for PARP2 in PARylating NRF2. In good agreement with that, pharmacological PARP inhibition by PJ34 reduced the nuclear localizaiton of NRF2 in control cells, while PARP inhibitors did not affect NRF2 localization in shPARP2 cells (Fig. 3B).

**Figure 4:**
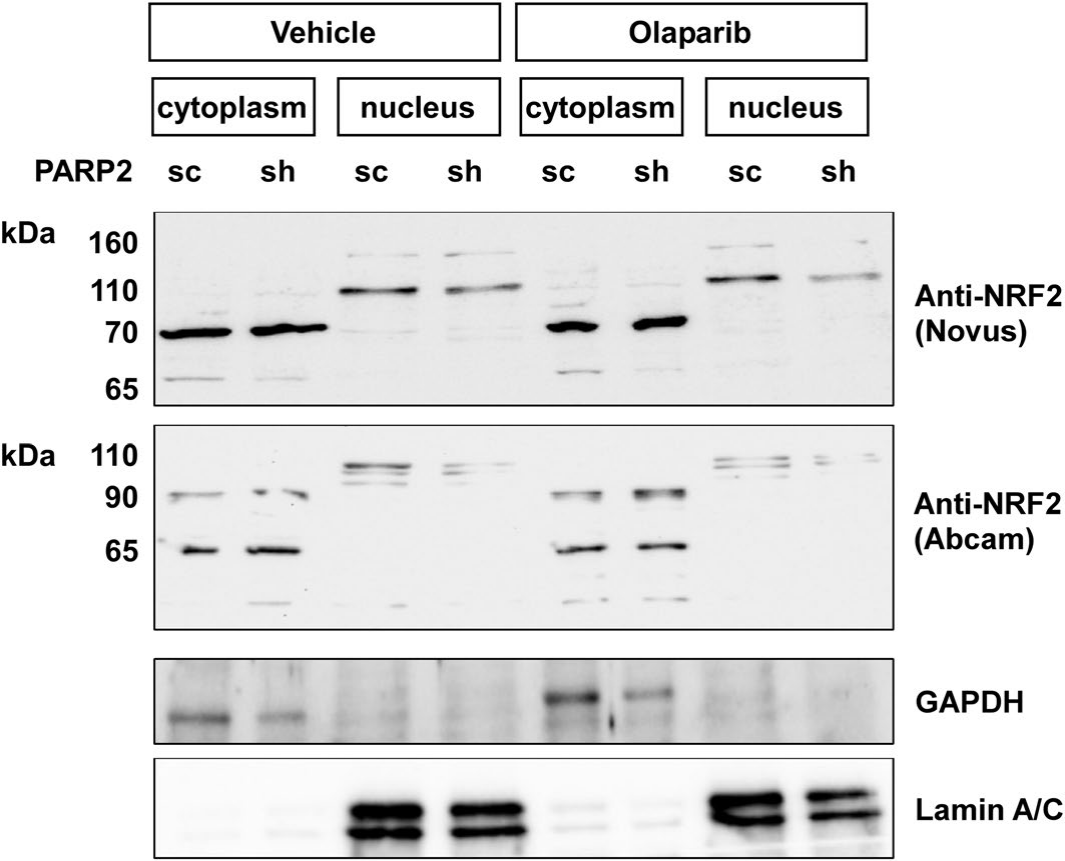
Lower nuclear NRF2 is detected in shPARP2 C2C12 cells. 1.5 × 10^6^ sc/shPARP2 C2C12 were seeded in Petri dishes; a set of cells were treated with 1 µM olaparib for 24 h. Then cells were harvested by scraping and cells were fractionated. Protein from the indicated fractions were separated by SDS-PAGE and the separated proteins were blotted. Blots were developed with the antibodies indicated.

To verify these findings with an alternative method, we performed cell fractionation based on the Sanders salt fractionation tachnique^67^. Fractionation was verified using antibodies against glyceraldehyde-3-phosphate-dehydrogenase (GAPDH) and Lamin A/C. GAPDH was detected only in the cytosol, while Lamin A/C was detected only in the nucleus (Fig. 4), verifying the specificity of the fractionation experiment. NRF2 signal was detected in both the cytosolic and the nuclear fraction, however, the apparent molecular weight of the signal was different in the compartments. The apparent molecular weight was higher in the nucleus (~ 110 kDa and ~ 160 kDa) than in the cytosol (~ 65 kDa and ~ 90 kDa) (Fig. 4). The ~ 110 kDa form was mostly detected in the cytosol, while the smaller isoforms were rather specific for the cytosol (Fig. 4). The cytosolic NRF2 signal increases, while the nuclear NRF2 signal decreases when PARP2 is silenced or olaparib is applied that points towards decreases to the nucleus-to-cytoplasm ratio of NRF2.

### Silencing of PARP2 leads to the repression of NRF2-dependent genes

**Figure 5:**
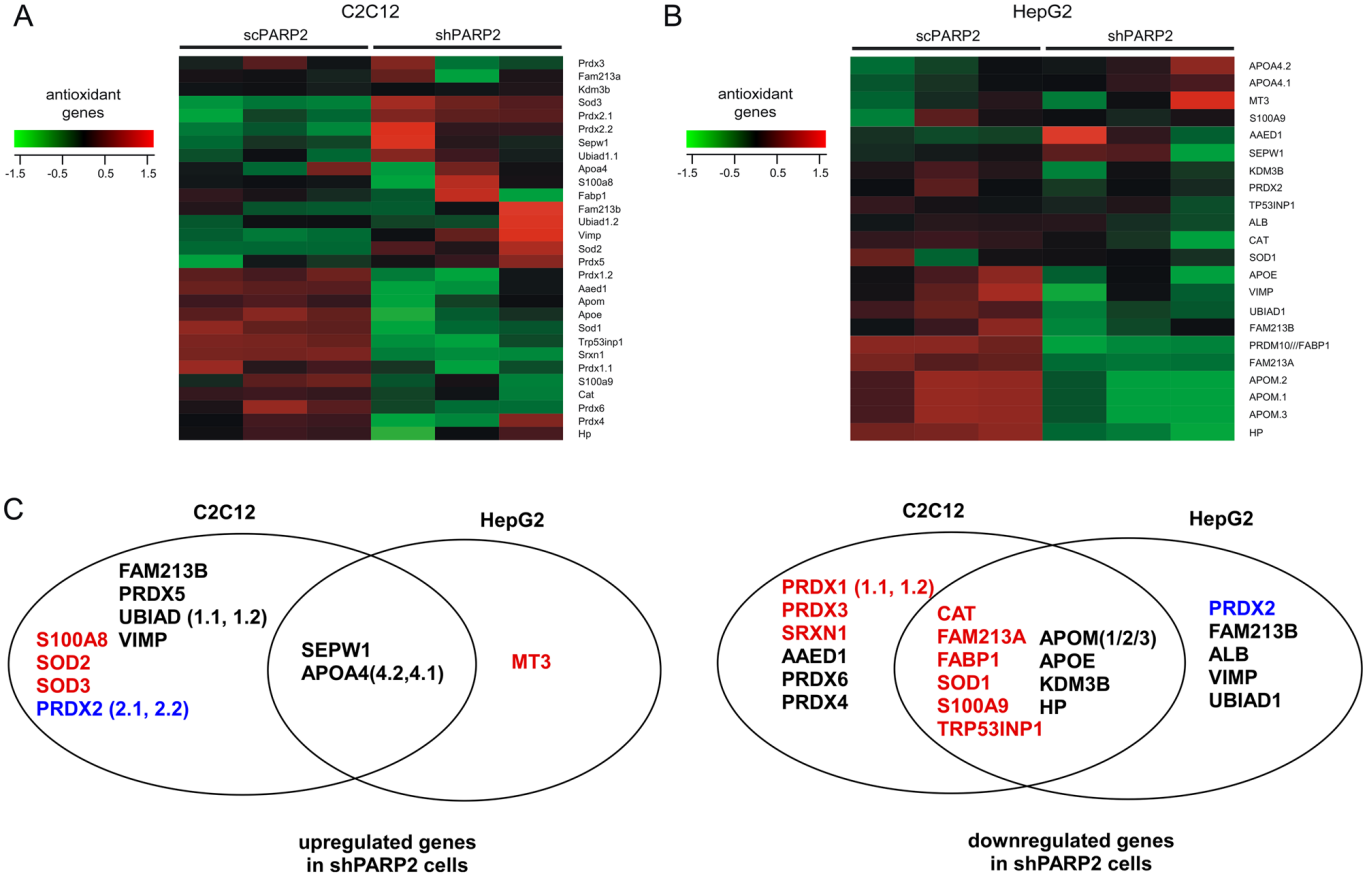
Silencing of PARP2 reorganizes the expression of antioxidant genes. (**A**,**B**) The expression of genes involved in antioxidant defense were assessed by the reanalysis of microarrays assessing (**A**) scPARP2 and shPARP2 C2C12 and (**B**) scPARP2 and shPARP2 HepG2 cells. (**C**) The differentially expressed redox genes identified upon the reanalysis of the microarrays were grouped as a function of the expression changes and were depicted in Venn-diagrams. In black, the NRF2-independent genes, in red the NRF2-dependent genes with similar changes between the microarray experiments. In blue, the NRF2-dependent genes with different expression between the microarray experiments.

We turned to decipher the possible consequences of the interaction between PARP2 and NRF2. To that end we reanalyzed a microarray experiment performed on scPARP2 and shPARP2 C2C12 cells^60^ and another, assessing gene expression changes between scPARP2 and shPARP2 HepG2 cells^68^. We assessed changes to the expression of genes with antioxidant function as described in the Materials and Methods section. Silencing of PARP2 reduced the expression of a set of antioxidant genes roughly comprising the half of all antioxidant genes assessed in both settings, while the remainder of the antioxidant genes were upregulated (Fig. 5A,B). Changes were in the same magnitude as in the case of the RT-qPCR study (± 1.5–twofold).

We grouped the up and downregulated antioxidant genes in both cell lines, assessed which genes are regulated anonimously between the cell lines and identified NRF2-dependent genes through literature search. Those genes were considered NRF2-dependent the expression of which was regulated by NRF2, those that are NRF2 partners or only modulated NRF2 activity be interfering with redox homeostasis (e.g. antioxidants as albumin) were not considered NRF2-dependent. Among the differentially regulated genes catalase (CAT)^69^, fatty acid binding protein 1 (FABP1)^70^, Family With Sequence Similarity 213, Member A (FAM213A)^71^, metallothionein (MT(3))^72^, peroxiredoxin 1 (PRDX1)^35^, peroxiredoxin 2 (PRDX2)^73^, peroxiredoxin 3 (PRDX3)^74,75^, superoxide dismutase 1 (SOD1)^76^, superoxide dismutase 2 (SOD2)^77^, superoxide dismutase 3 (SOD3)^78^, sulforedoxin 1 (SRXN1)^35^, S100A8^79^, S100A9^79^ and Tumor protein p53-inducible nuclear protein 1 (TRP53INP1)^80^ were identified as NRF2-dependent genes. Out of these, CAT, FAM213A, FABP1, SOD1, S100A9 and TRP53INP1 were downregulated both in shPARP2 C2C12 cells and shPARP2 HepG2 cells, while PRDX1, PRDX3 and SRXN1 were downregulated only in C2C12 cells (Fig. 5C). The expression of PRDX2 was differentially regulated between the two cell models (Fig. 5C). S100A8, SOD2 and SOD3 were upregulated in shPARP2 C2C12 cells and MT3 in shPARP2 HepG2 cells (Fig. 5C). Taken together, out of the the identified NRF2-dependent genes 9 were downregulated and 4 were upregulated.

## Discussion

**Figure 6:**
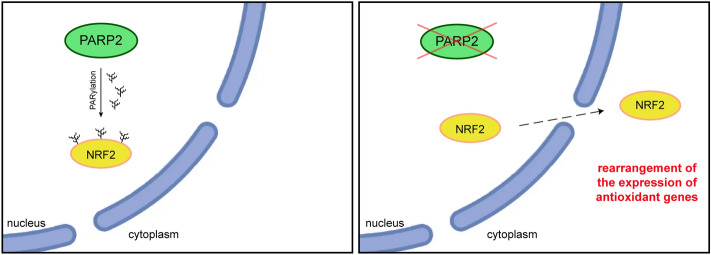
PARP2 PARylates NRF2 and traps it in the nucleus.

PARP2 is a member of the PARP family and has multiple functions overlapping with PARP1, however, clearly has individual functions independent of PARP1^19,20^. In this study we showed that PARP2 activity regulates NRF2 location and changes the pattern of NRF2-dependent gene expression. PARP2 can poly(ADP-ribosyl)ate NRF2 and tether it to the nucleus that is needed for the appropriate activity of NRF2. In contrast, when PARP2-dependent PARylation is absent, the cellular localization pattern of NRF2 changes, a larger fraction of NRF2 can be found in the cytoplasm. This observation suggests that NRF2 can over time translocate to the cytoplasm more easily (Fig. 6).

We observed changes to the expression of antioxidant genes upon the silencing of PARP2 and identified changes to the expression of 13 NRF2-dependent genes. Out of the 13 NRF2-dependent genes, 9 were suppressed, while 4 were upregulated in the absence of PARP2. Changes to gene expression therefore is linked to exhanges to cellular distribution of NRF2, however may not be the only explanation. PARylated NRF2 may have different transactivation properties than the non-PARylated form influencing transcription initiation efficiency and, subsequently, the NRF2-dependent transcriptome.

PARP enzymes were long-known to be involved in regulating gene expression^81–84^, and along that, the data on the involvement of PARP2 in the regulation of gene expression is accumulating^8,23,60–62,68,85–90^. In this study we identified a new PARP2-mediated transcription factor, NRF2. Prior, PARP2 was known to act as a transcriptional cofactor of nuclear receptors^91^, to modulate the mRNA expression of transcription factors (e.g. SIRT1 or SREBPs)^8,23,60,61,68^ or to impact on coenzymes of transcription factors (e.g. NAD + for SIRT1)^61^. However, this is the first time to show that PARP2 can regulate the activity of a transcription factor by affecting their cytosolic location. As pharmacological PARP inhibition reduces the nuclear fraction of NRF2, we suggest that the activity of PARP2 is necessary for the nuclear translocation or nuclear trapping of NRF2. On a broader scale we can deduct that NRF2 may be responsive to nuclear PARylation and, hence, act as a PARylation sensor. It is of note that PARP1 cannot poly(ADP-ribosyl)ate NRF2^53^, therefore, the pharmacological inhibition of PARP1 is unlikely to explain the cytosolic trapping/translocation of NRF2. The large negative charge of the PAR polymers may be a likely explanation for the restriction of active NRF2 to the nucleus. Currently interactions between PARP1 and NRF2 were shown in breast cancer cells^53^ and were suggested in inflammatory processes^92^ and bone formation^93^.

What could be the physiological relevance of our findings? As PARP2 can be activated by DNA strand breaks^7,9,10,94–99^ that are frequently induced by oxidative stress, hence, PARP2 activation in these situations can enhance and support NRF2 activation and antioxidant defense. Furthermore, We have previously detected increased oxidative stress in PARP2-silenced cells^27^ a portion of which stems from the mitochondria. Apparently, changes to antioxidant defense and the deregulation of NRF2 activity may also contribute to oxidative stress in the absence of PARP2.

Our findings may have implications in the PARP2-elicited adverse effects of pharmacological PARP inhibition^86,87^. Furthermore, our previous report^27^ provided evidence that the production of reactive species is needed for mitochondrial biogenesis in the absence of PARP2. In addition, changes to cellular redox balance play role in inflammation, cardiovascular and neoplastic diseases or aging, in which NRF2^52,100–103^ and PARP enzymes^1–3,28,29,104–109^. These processes are all characterized by PARP activation, more importantly, PARP2 activation was evidenced^8,89,110,111^, suggesting a broad applicability for our findings.

## Methods

### Chemicals

All chemicals, including PJ34, were from Sigma-Aldrich (St. Louis, MO, USA) unless stated otherwise.

### Cell culture

PARP2-silenced C2C12 cells were described in^23^. The 5′-AAGATGATGCCCAGAGGAACT-3′ was used as PARP2-specific interfering sequence, while the 5′-TTCGGGGAACAAACGTGCAAC-3′ sequence was used as a non-specific control^62^. C2C12 cells were cultured in DMEM (cat. no. D6429, Sigma-Aldrich, St. Louis, MO, USA) containing 10% FBS, 1% penicillin/streptomycin and 2 mM L-glutamine at 37 °C with 5% CO_2_. PARP2 was silenced by specific shRNA that was maintained over extended periods by selection under 2.5 µg/ml of puromycin. Cells containing the control, non-specific sequence are termed scPARP2, while those containing the PARP2-specific shRNA are termed shPARP2 cells.

### Immunofluorescence

Cells were seeded on glass coverslips into a 24-well plate and treated with 3 µM PJ34 for 24 h. After treatment cells were washed with PBS, fixed with 4% paraformaldehyde for 10 min at 37 °C and permeabilized with 1% Triton X-100 in PBS for 10 min. Between each steps, cells were washed three times with PBS. Cells were blocked with 1% BSA in PBS for 1 h at room temperature and incubated with primary antibody diluted in blocking buffer for overnight at 4 °C. Coverslips were washed three times with PBS and cells were incubated with the secondary antibody for 1 h at room temperature. Cell nuclei were visualized with DAPI (NucBlue Fixed Cell ReadyProbes Reagent, cat. no. R37606, Invitrogen).

Confocal images were acquired with Leica TCS SP8 confocal microscope (Leica) and LAS X 3.5.5.19976 software (Leica). Nonspecific binding of the secondary antibodies was checked in control experiments (data not shown). Antibodies are listed in Table 1.

**Table 1:** Antibodies used in immunofluorescence experiments.

Antibody	Company	Dilution
NRF2	Abcam, ab31163	1:100
NRF2	Novus Biologicals, NBP1-32,822	1:100

Processed images were analyzed using ImageJ software, NRF2 signal intensity nucleus/cell was determined.

### Total RNA preparation and reverse transcription-coupled quantitative PCR (RT-qPCR)

Total RNA from cells was prepared using TRIzol reagent (cat. no. 15596018, Invitrogen, Carlsbad, CA, USA) according to the manufacturer’s instructions. DNase treatment of total RNA samples was performed using 2U DNaseI (cat.no A2222, Thermo Fischer Scientific, Waltham, MA, USA) per 10 µg RNA. 2 µg of total RNA were used for reverse transcription using High-Capacity cDNA Reverse Transcription Kit (cat. no. 4368814, Applied Biosystems, Foster City, CA, USA) according to the manufacturer’s instructions. The reverse transcription reaction mixtures were supplemented with RNase inhibitor (cat.no 8080119, Applied Biosystems, Foster City, CA, USA). The qPCR reactions were performed with qPCRBIO SyGreen Lo-ROX Supermix (PCR Biosystems Ltd., London, UK) using 20 ng diluted cDNA with the appropriate primers at 500 nM final concentration in 10 µl total volume. Amplification reactions were performed using a Light-Cycler 480 system (Roche Applied Science, Basel, Switzerland). Cycling conditions are given in Table 2. Expression was normalized to the geometric mean of murine 36B4 and cyclophilin values. Primers are listed in Table 3.

**Table 2:** qPCR cycling conditions.

	Temperature	Time	Cycles
Polymerase activation	95 °C	10 min	1
Denaturation	95 °C	10 s	40
Annealing/Extension	62 °C	20 s	
Melting curve analysis	95 °C	5 s	1
	65 °C	1 min	
	65 °C to 97 °C	ramp rate 0.11 °C/s	
	40 °C	30 s

**Table 3:** Primer sets used in the study. Primers bore no modification.

Gene name	Primers (5′-3′)	Amplicon length (bp)	Location	Location	Specificity check	Splice variants targeted	Calibration curve equation, R^2^	Efficiency
KEAP1 NM_001110305	F: CAGCAGCGTGGAGAGATATG R: GGTTAGTCCCGTCAAAGCCC	137	exonic	fw 1898–1917 rev 2034–2015	BLAST	yes (1,2,3,4)	y = 3.608x + 29.03 Error = 0.0624	1.893
MAF K NM_010757	F: GGCAGGGACTTGTTGTTCTTC R: CGCCTCCTTCTTGACCTTCAAT	246	exonic	fw 27–47 rev 272–251	BLAST	-	y = 3.517x + 30.87 Error = 0.0312	1.924
MAF G NM_010756	F: TGAGTGCCTGCTCACTGTGTC R: TGGCTCCCGCTTCACCTTTA	81	exonic	fw 185–205 rev 265–246	BLAST	-	y = 3.436x + 27.94 Error = 0.0565	1.955
Nrf2 NM_010902.5	F: CATCAGGCCCAGTCCCTCAAT R: CAGCGGTAGTATCAGCCAGCT	157	intronic	fw 565–585 rev 721–701	BLAST	-	y = 3.508x + 26.66 Error = 0.0211	1.928
Parp2 NM_009632	F: GGAAGGCGAGTGCTAAATGAA R: AAGGTCTTCACAGAGTCTCGATTG	167	intronic	fw 63–83 rev 229–206	BLAST	-	y = 3.724x + 31.47 Error = 0.112	1.856
cyclophilin NM_011149	F: TGGAGAGCACCAAGACAGACA R: TGCCGGAGTCGACAATGAT	66	intronic	fw 642–662 rev 707–689	BLAST	-	y = 3.428x + 25.48 Error = 0.0514	1.958
36B4 NM:007,475.5	F: AGATTCGGGATATGCTGTTGG R: AAAGCCTGGAAGAAGGAGGTC	133	intronic	fw 411–431 rev 543–523	BLAST	-	y = 3.460x + 21.34 Error = 0.0459	1.946

RNA integrity was not verified. Specificity was assessed by assessing the melting curve, those well that contained more than one peak were omitted. Results were assessed using the software of the Lighcycler II instrument (version 1.5.0) Outliers were not assessed and no values were rejected. C_T_ was identified using the second derivative method provided by the Lighcycler II software. Three technical replicates were used. We reported the results according to the MIQE guidelines^112^.

### Immunoprecipitation

Cells were washed with PBS and lysed in lysis buffer (50 mM Tris, pH 8, 150 mM NaCl, 1% Triton X-100, 0.5% sodium deoxycholate, 0.1% SDS, 1 mM EDTA, 1 mM Na_3_VO_4_, 1 mM NaF, 1 mM PMSF, protease inhibitor cocktail) on ice. 20–20 µl of Protein A-Protein G Sepharose beads were resuspended in immunoprecipitation buffer (20 mM Tris, 150 mM NaCl, 2 mM EDTA, 1% NP-40, protease inhibitor cocktail). Samples were precleared for 1 h, the primary antibody was added to the precleared samples for 2 h, then beads were added to the samples for overnight at 4 °C with gentle rotation. The resulting immunoprecipitates were washed with TBS_Tween_, boiled with 50 µl 5 × SDS sample buffer (310 mM Tris–HCl, pH 6.8, 50% glycerol, 10% SDS, 100 mM DTT, 0.01% bromophenol blue) and 2-mercaptoethanol and used for Western blotting.

### Cell fractionation

1.5 × 10^6^ sc/shPARP2 C2C12 cells were grown on 10 cm Petri dishes to 80% confluency, washed and scraped in ice-cold PBS, then pelleted by centrifugation at 4 °C at 1,500 × g for 3 min. After removing PBS, cells were homogenized in 1800 μl buffer A (10 mM Hepes pH 7.9, 10 mM KCl, 0.1 mM EDTA, 0.1 mM EGTA, 0.5% NP40, 1 mM DTT, 1 × protease inhibitor cocktail, 1 mM NaF, 1 mM PMSF, 1 mM Na_3_VO_4_) by suspending with a 26-gauge needle 10 times using a syringe. The lysate was vortexed for 15 s, then centrifuged at 4 °C at 16,000 × g for 1 min. The resulting supernatant was used as cytosolic fraction. The pellet was then washed in 1000 μl buffer A by passing through a 26G needle 10 times. After centrifugation (4 °C at 16,000 × g for 1 min) 1200 μl buffer B (20 mM Hepes pH 7.9, 420 mM NaCl, 0.5 mM EDTA, 0.5 mM EGTA, 1 mM DTT, 1 × protease inhibitor cocktail, 1 mM NaF, 1 mM PMSF, 1 mM Na_3_VO_4_) was added to the pellet and it was incubated for 25 min on ice, while being vortexed several times. Then the pellet was sonicated (3 times for 10 s), centrifuged (4 °C at 12 000 × g for 10 min) and used as nuclear fraction.

### SDS-PAGE and western blotting

Cells were washed with PBS, collected and lysed in lysis buffer (50 mM Tris, pH 8, 150 mM NaCl, 1% Triton X-100, 0.5% sodium deoxycholate, 0.1% SDS, 1 mM EDTA, 1 mM Na_3_VO_4_, 1 mM NaF, 1 mM PMSF, protease inhibitor cocktail) on ice and boiled with 5 × SDS sample buffer (310 mM Tris–HCl, pH 6.8, 50% glycerol, 10% SDS, 100 mM DTT, 0.01% bromophenol blue) and 2-mercaptoethanol. Protein extracts were separated by SDS-PAGE on 8% acrylamide gels and transferred onto nitrocellulose membranes. Membranes were blocked with 5% BSA in TBS_Tween_ for 1 h at room temperature and incubated with primary antibodies overnight at 4 °C. Membranes were probed with the respective peroxidase-conjugated secondary antibody. Signals were visualized by enhanced chemiluminescence reaction and captured by ChemiDoc Touch Imaging System (Bio-Rad, Hercules, CA, USA). Bands were quantified by densitometry using the ImageJ software^113^ and densitometry data were analyzed by statistical methods. Primary and secondary antibodies are listed in Table 4. The original description of the method can be found in^114^.

**Table 4:** Antibodies used in Western blot experiments.

Antibody	Company	Dilution
GAPDH	Santa Cruz, sc-47724	1:10 000
LAMIN A/C	Santa Cruz, sc-20681	1:2000
NRF2	Abcam, ab31163	1:1000
NRF2	Novus Biologicals, NBP1-32,822	1:1000
PARP-2	Enzo Life Sciences, ALX-210–899-R100	1:2000
Poly(ADP-ribose) (PAR)	Enzo Life Sciences, BML-SA216-0100	1:1000
Anti-mouse IgG, HRP-linked	Sigma-Aldrich, A9044	1:2000
Anti-rabbit IgG, HRP-linked	Cell Signaling Technology, 7074	1:2000
Anti-β-actin − Peroxidase	Sigma-Aldrich, A3854	1:20,000

### Microarray reanalysis

The expression patterns were analyzed on samples from two microarray experiments with PARP-2 silencing: one was conducted on human HepG2 cells^68^ (GEO accession number: GSE43981), the other was on mouse C2C12 cells^60^ (GEO accession number: GSE108737).

For both mouse and human, four groups of genes have been constructed from the GO database^115,116^ (www.geneontology.com). To obtain the expression of antioxidant genes, genes were extracted based on their labeling as ‘antioxidant’. The superoxide dismutase genes (SOD1, SOD2), originally labelled as ‘reactive oxygen species biosynthetic process’, were added to the antioxidant lists as the products of these genes are known antioxidants^117^.

We used the GEOquery R package^118^ to retrieve and parse the raw data from both experiments. Both experiments had 3–3 replicates of controls and PARP-2 silenced samples. The limma package^119^ was used to calculate the log2 expression values and to compare the controls and the silenced samples, and calculate the differential expressions. A least squares linear model was fit, followed by an empirical Bayes model and Benjamini & Hochberg correction for multiple comparisons to get the fold change, moderated F and t-statistics, log-odds of differential expression and the corrected p-values for each gene or gene set represented by the Affymetrix IDs. These complete lists were filtered to get only the genes contained in the predefined antioxidant list.

The Affymetrix IDs were converted to gene symbols, taking into consideration that sometimes an Affymetrix probe represents multiple genes, and sometimes a gene is represented by multiple probes. In the former case, the multiple gene symbols separated with the symbol “///” are shown in a single line corresponding to the probe; in the latter case, the identical gene symbols across multiple lines are indexed as “0.1”, “0.2” and so on.

Heatmaps were generated with the limma package as well, using the log2 expression values of each sample.

### Statistical analysis

Statistical analyses, except for the microarray reanalysis, were performed with Prism software v8 (GraphPad Software, San Diego, CA, USA). All groups were checked for normal distribution using the D’Agostino-Pearson method. For comparing two groups, two-sided Student’s tests were applied. For multiple comparisons, one-way ANOVA tests were conducted, followed by Tukey’s post hoc test (to compare all possible combinations). The levels of significance are indicated in figure captions. The *n* number denotes the number of the biological replicates.

## Supplementary Information

Supplementary Information.

## Data Availability

Primary data and full blot images related to the study are available at https://figshare.com/s/a378d2bf46f1969819eb (https://doi.org/10.6084/m9.figshare.17127008). Fullblot images are uploaded as supplementary material.

## References

[1] Virag L, Szabo E, Bakondi E, Bai P, Gergely P, Hunyadi J (2002). Nitric oxide-peroxynitrite-poly(ADP-ribose) polymerase pathway in the skin. Exp. Dermatol..

[2] Pacher P, Beckman JS, Liaudet L (2007). Nitric oxide and peroxynitrite in health and disease. Physiol. Rev..

[3] Pacher P, Szabo C (2008). Role of the peroxynitrite-poly(ADP-ribose) polymerase pathway in human disease. Am. J. Pathol..

[4] Virag L, Scott GS, Cuzzocrea S, Marmer D, Salzman AL, Szabo C (1998). Peroxynitrite-induced thymocyte apoptosis: the role of caspases and poly (ADP-ribose) synthetase (PARS) activation. Immunology.

[5] Virag L, Szabo C (2002). The therapeutic potential of poly(ADP-ribose) polymerase inhibitors. Pharmacol. Rev..

[6] Lüscher B, Ahel I, Altmeyer M, Ashworth A, Bai P, Chang P (2021). ADP-ribosyltransferases, an update on function and nomenclature. FEBS J..

[7] Beck C, Robert I, Reina-San-Martin B, Schreiber V, Dantzer F (2014). Poly(ADP-ribose) polymerases in double-strand break repair: focus on PARP1, PARP2 and PARP3. Exp. Cell Res..

[8] Szanto M, Rutkai I, Hegedus C, Czikora A, Rozsahegyi M, Kiss B (2011). Poly(ADP-ribose) polymerase-2 depletion reduces doxorubicin-induced damage through SIRT1 induction. Cardiovasc. Res..

[9] Schreiber V, Ame JC, Dolle P, Schultz I, Rinaldi B, Fraulob V (2002). Poly(ADP-ribose) polymerase-2 (PARP-2) is required for efficient base excision DNA repair in association with PARP-1 and XRCC1. J. Biol. Chem..

[10] Ame JC, Rolli V, Schreiber V, Niedergang C, Apiou F, Decker P (1999). PARP-2, A novel mammalian DNA damage-dependent poly(ADP-ribose) polymerase. J. Biol. Chem..

[11] Pic E, Gagne JP, Poirier GG (2010). Mass spectrometry-based functional proteomics of poly(ADP-ribose) polymerase-1. Expert Rev. Proteom..

[12] Palazzo L, Leidecker O, Prokhorova E, Dauben H, Matic I, Ahel I (2018). Serine is the major residue for ADP-ribosylation upon DNA damage. Elife.

[13] Crawford K, Bonfiglio JJ, Mikoc A, Matic I, Ahel I (2018). Specificity of reversible ADP-ribosylation and regulation of cellular processes. Crit. Rev. Biochem. Mol. Biol..

[14] Jungmichel S, Rosenthal F, Altmeyer M, Lukas J, Hottiger MO, Nielsen ML (2013). Proteome-wide identification of poly(ADP-Ribosyl)ation targets in different genotoxic stress responses. Mol. Cell.

[15] Abplanalp J, Leutert M, Frugier E, Nowak K, Feurer R, Kato J (2017). Proteomic analyses identify ARH3 as a serine mono-ADP-ribosylhydrolase. Nat. Commun..

[16] Leutert M, Menzel S, Braren R, Rissiek B, Hopp AK, Nowak K (2018). Proteomic characterization of the heart and skeletal muscle reveals widespread arginine ADP-ribosylation by the ARTC1 ectoenzyme. Cell Rep..

[17] Leslie Pedrioli DM, Leutert M, Bilan V, Nowak K, Gunasekera K, Ferrari E (2018). Comprehensive ADP-ribosylome analysis identifies tyrosine as an ADP-ribose acceptor site. EMBO Rep..

[18] Bai P (2015). Biology of poly(ADP-Ribose) polymerases: The factotums of cell maintenance. Mol. Cell.

[19] Szanto M, Brunyánszki A, Kiss B, Nagy L, Gergely P, Virag L (2012). Poly(ADP-ribose) polymerase-2: Emerging transcriptional roles of a DNA repair protein. Cell. Mol. Life Sci..

[20] Yelamos J, Schreiber V, Dantzer F (2008). Toward specific functions of poly(ADP-ribose) polymerase-2. Trends Mol. Med..

[21] Kraus WL, Lis JT (2003). PARP goes transcription. Cell.

[22] Kofler J, Otsuka T, Zhang Z, Noppens R, Grafe MR, Koh DW (2006). Differential effect of PARP-2 deletion on brain injury after focal and global cerebral ischemia. J. Cereb. Blood Flow Metab..

[23] Bai P, Canto C, Brunyánszki A, Huber A, Szántó M, Cen Y (2011). PARP-2 regulates SIRT1 expression and whole-body energy expenditure. Cell Metab..

[24] Li X, Klaus JA, Zhang J, Xu Z, Kibler KK, Andrabi SA (2010). Contributions of poly(ADP-ribose) polymerase-1 and -2 to nuclear translocation of apoptosis-inducing factor and injury from focal cerebral ischemia. J. Neurochem..

[25] Spina-Purrello V, Giliberto S, Barresi V, Nicoletti VG, Giuffrida Stella AM, Rizzarelli E (2011). Modulation of PARP-1 and PARP-2 Expression by L-carnosine and trehalose after LPS and INFgamma-induced oxidative stress. Neurochem. Res..

[26] Mateu-Jimenez M, Cucarull-Martinez B, Yelamos J, Barreiro E (2016). Reduced tumor burden through increased oxidative stress in lung adenocarcinoma cells of PARP-1 and PARP-2 knockout mice. Biochimie.

[27] Jankó L, Kovács T, Laczik M, Sári Z, Ujlaki G, Kis G (2021). Silencing of poly(ADP-Ribose) polymerase-2 induces mitochondrial reactive species production and mitochondrial fragmentation. Cells.

[28] Curtin N, Szabo C (2013). Therapeutic applications of PARP inhibitors: Anticancer therapy and beyond. Mol. Aspects Med..

[29] Curtin NJ, Szabo C (2020). Poly(ADP-ribose) polymerase inhibition: Past, present and future. Nat. Rev. Drug Discovery.

[30] Curtin N, Bányai K, Thaventhiran J, Le Quesne J, Helyes Z, Bai P (2020). Repositioning PARP inhibitors for SARS-CoV-2 infection (COVID-19); A new multi-pronged therapy for acute respiratory distress syndrome?. Br. J. Pharmacol..

[31] Wahlberg E, Karlberg T, Kouznetsova E, Markova N, Macchiarulo A, Thorsell AG (2012). Family-wide chemical profiling and structural analysis of PARP and tankyrase inhibitors. Nat. Biotechnol..

[32] Bochum S, Berger S, Martens UM (2018). Olaparib. Recent Results Cancer Res..

[33] Deeks ED (2015). Olaparib: First global approval. Drugs.

[34] Nguyen T, Nioi P, Pickett CB (2009). The Nrf2-antioxidant response element signaling pathway and its activation by oxidative stress. J. Biol. Chem..

[35] Malhotra D, Portales-Casamar E, Singh A, Srivastava S, Arenillas D, Happel C (2010). Global mapping of binding sites for Nrf2 identifies novel targets in cell survival response through ChIP-Seq profiling and network analysis. Nucl. Acids Res..

[36] Venugopal R, Jaiswal AK (1996). Nrf1 and Nrf2 positively and c-Fos and Fra1 negatively regulate the human antioxidant response element-mediated expression of NAD(P)H:quinone oxidoreductase1 gene. Proc. Natl. Acad. Sci. USA.

[37] Itoh K, Wakabayashi N, Katoh Y, Ishii T, Igarashi K, Engel JD (1999). Keap1 represses nuclear activation of antioxidant responsive elements by Nrf2 through binding to the amino-terminal Neh2 domain. Genes Dev..

[38] Nguyen T, Sherratt PJ, Huang HC, Yang CS, Pickett CB (2003). Increased protein stability as a mechanism that enhances Nrf2-mediated transcriptional activation of the antioxidant response element: Degradation of Nrf2 by the 26 S proteasome. J. Biol. Chem..

[39] Tebay LE, Robertson H, Durant ST, Vitale SR, Penning TM, Dinkova-Kostova AT (2015). Mechanisms of activation of the transcription factor Nrf2 by redox stressors, nutrient cues, and energy status and the pathways through which it attenuates degenerative disease. Free Radical Biol. Med..

[40] de la Vega MR, Chapman E, Zhang DD (2018). NRF2 and the Hallmarks of Cancer. Cancer Cell.

[41] Dinkova-Kostova AT, Holtzclaw WD, Cole RN, Itoh K, Wakabayashi N, Katoh Y (2002). Direct evidence that sulfhydryl groups of Keap1 are the sensors regulating induction of phase 2 enzymes that protect against carcinogens and oxidants. PNAS (Proc. Natl. Acad. Sci. USA)..

[42] McMahon M, Lamont DJ, Beattie KA, Hayes JD (2010). Keap1 perceives stress via three sensors for the endogenous signaling molecules nitric oxide, zinc, and alkenals. PNAS (Proc. Natl. Acad. Sci. USA).

[43] He X, Ma Q (2010). Critical cysteine residues of Kelch-like ECH-associated protein 1 in arsenic sensing and suppression of nuclear factor erythroid 2-related factor 2. J. Pharmacol. Exp. Ther..

[44] Yamamoto T, Suzuki T, Kobayashi A, Wakabayashi J, Maher J, Motohashi H (2008). Physiological significance of reactive cysteine residues of Keap1 in determining Nrf2 activity. Mol. Cell. Biol..

[45] Magesh S, Chen Y, Hu L (2012). Small molecule modulators of Keap1-Nrf2-ARE pathway as potential preventive and therapeutic agents. Med. Res. Rev..

[46] He X, Ma Q (2009). NRF2 cysteine residues are critical for oxidant/electrophile-sensing, Kelch-like ECH-associated protein-1-dependent ubiquitination-proteasomal degradation, and transcription activation. Mol. Pharmacol..

[47] Hirotsu Y, Katsuoka F, Funayama R, Nagashima T, Nishida Y, Nakayama K (2012). Nrf2-MafG heterodimers contribute globally to antioxidant and metabolic networks. Nucl. Acids Res..

[48] Venugopal R, Jaiswal AK (1998). Nrf2 and Nrf1 in association with Jun proteins regulate antioxidant response element-mediated expression and coordinated induction of genes encoding detoxifying enzymes. Oncogene.

[49] McMahon M, Thomas N, Itoh K, Yamamoto M, Hayes JD (2004). Redox-regulated turnover of Nrf2 is determined by at least two separate protein domains, the redox-sensitive Neh2 degron and the redox-insensitive Neh6 degron. J. Biol. Chem..

[50] Zhu M, Fahl WE (2001). Functional characterization of transcription regulators that interact with the electrophile response element. Biochem. Biophys. Res. Commun..

[51] Tonelli C, Chio IIC, Tuveson DA (2018). Transcriptional Regulation by Nrf2. Antioxid. Redox Signal..

[52] Ungvari Z, Tarantini S, Nyul-Toth A, Kiss T, Yabluchanskiy A, Csipo T (2019). Nrf2 dysfunction and impaired cellular resilience to oxidative stressors in the aged vasculature: from increased cellular senescence to the pathogenesis of age-related vascular diseases. Geroscience..

[53] Wu T, Wang XJ, Tian W, Jaramillo MC, Lau A, Zhang DD (2014). Poly(ADP-ribose) polymerase-1 modulates Nrf2-dependent transcription. Free Radical Biol. Med..

[54] Hendriks IA, Larsen SC, Nielsen ML (2019). An Advanced Strategy for Comprehensive Profiling of ADP-ribosylation Sites Using Mass Spectrometry-based Proteomics. Mol. Cellular Proteom.: MCP..

[55] Ahn B, Pharaoh G, Premkumar P, Huseman K, Ranjit R, Kinter M (2018). Nrf2 deficiency exacerbates age-related contractile dysfunction and loss of skeletal muscle mass. Redox Biol..

[56] Tarantini S, Valcarcel-Ares MN, Yabluchanskiy A, Tucsek Z, Hertelendy P, Kiss T (2018). Nrf2 deficiency exacerbates obesity-induced oxidative stress, neurovascular dysfunction, blood-brain barrier disruption, neuroinflammation, amyloidogenic gene expression, and cognitive decline in mice, mimicking the aging phenotype. J. Gerontol. A Biol. Sci. Med. Sci..

[57] Fulop GA, Kiss T, Tarantini S, Balasubramanian P, Yabluchanskiy A, Farkas E (2018). Nrf2 deficiency in aged mice exacerbates cellular senescence promoting cerebrovascular inflammation. Geroscience..

[58] Vida A, Marton J, Miko E, Bai P (2017). Metabolic roles of poly(ADP-ribose) polymerases. Semin. Cell abd Dev. Biol..

[59] Bai P, Canto C (2012). The role of PARP-1 and PARP-2 enzymes in metabolic regulation and disease. Cell Metab..

[60] Marton J, Peter M, Balogh G, Bodi B, Vida A, Szanto M (2018). Poly(ADP-ribose) polymerase-2 is a lipid-modulated modulator of muscular lipid homeostasis. Biochimica et Biophysica Acta - Molecular and Cell Biology of Lipids..

[61] Mohamed JS, Hajira A, Pardo PS, Boriek AM (2014). MicroRNA-149 inhibits PARP-2 and promotes mitochondrial biogenesis via SIRT-1/PGC-1alpha network in skeletal muscle. Diabetes.

[62] Bai P, Houten SM, Huber A, Schreiber V, Watanabe M, Kiss B (2007). Poly(ADP-ribose) polymerase-2 controls adipocyte differentiation and adipose tissue function through the regulation of the activity of the retinoid X receptor/peroxisome proliferator-activated receptor-gamma heterodimer. J. Biol. Chem..

[63] Lau A, Tian W, Whitman SA, Zhang DD (2013). The predicted molecular weight of Nrf2: it is what it is not. Antioxid. Redox Signal..

[64] Venkatraman G, Benesch MG, Tang X, Dewald J, McMullen TP, Brindley DN (2015). Lysophosphatidate signaling stabilizes Nrf2 and increases the expression of genes involved in drug resistance and oxidative stress responses: implications for cancer treatment. FASEB J..

[65] Kovács P, Csonka T, Kovács T, Sári Z, Ujlaki G, Sipos A (2019). Lithocholic acid, a metabolite of the microbiome, increases oxidative stress in breast cancer. Cancers (Basel)..

[66] Tartier L, Spenlehauer C, Newman HC, Folkard M, Prise KM, Michael BD (2003). Local DNA damage by proton microbeam irradiation induces poly(ADP-ribose) synthesis in mammalian cells. Mutagenesis.

[67] Sanders MM (1978). Fractionation of nucleosomes by salt elution from micrococcal nuclease-digested nuclei. J Cell Biol..

[68] Szanto M, Brunyanszki A, Marton J, Vamosi G, Nagy L, Fodor T (2014). Deletion of PARP-2 induces hepatic cholesterol accumulation and decrease in HDL levels. Biochimica and Biophysica Acta - Molecular Basis of Disease..

[69] Zhu H, Itoh K, Yamamoto M, Zweier JL, Li Y (2005). Role of Nrf2 signaling in regulation of antioxidants and phase 2 enzymes in cardiac fibroblasts: protection against reactive oxygen and nitrogen species-induced cell injury. FEBS Lett..

[70] Chen XY, Li R, Geng ZY (2015). Cold stress initiates the Nrf2/UGT1A1/L-FABP signaling pathway in chickens. Poult. Sci..

[71] Oh CK, Ha M, Han ME, Heo HJ, Myung K, Lee Y (2020). FAM213A is linked to prognostic significance in acute myeloid leukemia through regulation of oxidative stress and myelopoiesis. Hematol. Oncol..

[72] Houessinon A, François C, Sauzay C, Louandre C, Mongelard G, Godin C (2016). Metallothionein-1 as a biomarker of altered redox metabolism in hepatocellular carcinoma cells exposed to sorafenib. Mol. Cancer.

[73] Li W, Febbraio M, Reddy SP, Yu DY, Yamamoto M, Silverstein RL (2010). CD36 participates in a signaling pathway that regulates ROS formation in murine VSMCs. J. Clin. Investig..

[74] Li L, Obinata M, Hori K (2010). Role of peroxiredoxin III in the pathogenesis of pre-eclampsia as evidenced in mice. Oxid. Med. Cell. Longev..

[75] Kim YS, Lee HL, Lee KB, Park JH, Chung WY, Lee KS (2011). Nuclear factor E2-related factor 2 dependent overexpression of sulfiredoxin and peroxiredoxin III in human lung cancer. Korean J. Intern. Med..

[76] Milani P, Ambrosi G, Gammoh O, Blandini F, Cereda C (2013). SOD1 and DJ-1 converge at Nrf2 pathway: A clue for antioxidant therapeutic potential in neurodegeneration. Oxid. Med. Cell. Longev..

[77] Reisman SA, Yeager RL, Yamamoto M, Klaassen CD (2009). Increased Nrf2 activation in livers from Keap1-knockdown mice increases expression of cytoprotective genes that detoxify electrophiles more than those that detoxify reactive oxygen species. Toxicol. Sci..

[78] Singh B, Bhat HK (2012). Superoxide dismutase 3 is induced by antioxidants, inhibits oxidative DNA damage and is associated with inhibition of estrogen-induced breast cancer. Carcinogenesis.

[79] Sumi D, Shimizu Y, Himeno S (2013). Involvement of Nrf2 activation in the upregulation of S100A9 by exposure to inorganic arsenite. IJMM..

[80] Nair S, Xu C, Shen G, Hebbar V, Gopalakrishnan A, Hu R (2007). Toxicogenomics of endoplasmic reticulum stress inducer tunicamycin in the small intestine and liver of Nrf2 knockout and C57BL/6J mice. Toxicol. Lett..

[81] Kraus WL, Hottiger MO (2013). PARP-1 and gene regulation: Progress and puzzles. Mol. Aspects Med..

[82] Oliver FJ, Menissier-de MJ, Nacci C, Decker P, Andriantsitohaina R, Muller S (1999). Resistance to endotoxic shock as a consequence of defective NF-kappaB activation in poly (ADP-ribose) polymerase-1 deficient mice. EMBO J..

[83] Ryu KW, Kim DS, Kraus WL (2015). New facets in the regulation of gene expression by ADP-ribosylation and poly(ADP-ribose) polymerases. Chem. Rev..

[84] Challa S, Khulpateea BR, Nandu T, Camacho CV, Ryu KW, Chen H (2021). Ribosome ADP-ribosylation inhibits translation and maintains proteostasis in cancers. Cell.

[85] Yelamos J, Monreal Y, Saenz L, Aguado E, Schreiber V, Mota R (2006). PARP-2 deficiency affects the survival of CD4+CD8+ double-positive thymocytes. EMBO J..

[86] Farres J, Martin-Caballero J, Martinez C, Lozano JJ, Llacuna L, Ampurdanes C (2013). PARP-2 is required to maintain hematopoiesis following sublethal gamma-irradiation in mice. Blood.

[87] Farres J, Llacuna L, Martin-Caballero J, Martinez C, Lozano JJ, Ampurdanes C (2015). PARP-2 sustains erythropoiesis in mice by limiting replicative stress in erythroid progenitors. Cell Death Differ..

[88] Chacon-Cabrera A, Fermoselle C, Salmela I, Yelamos J, Barreiro E (2015). MicroRNA expression and protein acetylation pattern in respiratory and limb muscles of Parp-1(-/-) and Parp-2(-/-) mice with lung cancer cachexia. Biochimica and Biophysica Acta..

[89] Navarro J, Gozalbo-Lopez B, Mendez AC, Dantzer F, Schreiber V, Martinez C (2017). PARP-1/PARP-2 double deficiency in mouse T cells results in faulty immune responses and T lymphomas. Sci. Rep..

[90] Maeda Y, Hunter TC, Loudy DE, Dave V, Schreiber V, Whitsett JA (2006). PARP-2 interacts with TTF-1 and regulates expression of surfactant protein-B. J. Biol. Chem..

[91] Szanto M, Gupte R, Kraus WL, Pacher P, Bai P (2021). PARPs in lipid metabolism and related diseases. Prog. Lipid Res..

[92] Pietrzak J, Spickett CM, Ploszaj T, Virag L, Robaszkiewicz A (2018). PARP1 promoter links cell cycle progression with adaptation to oxidative environment. Redox Biol..

[93] Hegedus C, Robaszkiewicz A, Lakatos P, Szabo E, Virag L (2015). Poly(ADP-ribose) in the bone: From oxidative stress signal to structural element. Free Radical Biol. Med..

[94] Chalmers A, Johnston P, Woodcock M, Joiner M, Marples B (2004). PARP-1, PARP-2, and the cellular response to low doses of ionizing radiation. Int. J. Radiat. Oncol. Biol. Phys..

[95] Langelier MF, Riccio AA, Pascal JM (2014). PARP-2 and PARP-3 are selectively activated by 5' phosphorylated DNA breaks through an allosteric regulatory mechanism shared with PARP-1. Nucl. Acids Res..

[96] Kutuzov MM, Khodyreva SN, Ilina ES, Sukhanova MV, Ame JC, Lavrik OI (2015). Interaction of PARP-2 with AP site containing DNA. Biochimie.

[97] Nakamoto MY, Rudolph J, Wuttke DS, Luger K (2019). Nonspecific binding of RNA to PARP1 and PARP2 does not lead to catalytic activation. Biochemistry.

[98] Obaji E, Haikarainen T, Lehtio L (2016). Characterization of the DNA dependent activation of human ARTD2/PARP2. Sci. Rep..

[99] Obaji E, Haikarainen T, Lehtio L (2018). Structural basis for DNA break recognition by ARTD2/PARP2. Nucl. Acids Res..

[100] Silva-Palacios A, Konigsberg M, Zazueta C (2016). Nrf2 signaling and redox homeostasis in the aging heart: A potential target to prevent cardiovascular diseases?. Ageing Res. Rev..

[101] Cuadrado A, Rojo AI, Wells G, Hayes JD, Cousin SP, Rumsey WL (2019). Therapeutic targeting of the NRF2 and KEAP1 partnership in chronic diseases. Nat. Rev. Drug Discovery.

[102] Yagishita Y, Gatbonton-Schwager TN, McCallum ML, Kensler TW (2020). Current landscape of NRF2 biomarkers in clinical trials. Antioxidants (Basel).

[103] Smolková K, Mikó E, Kovács T, Leguina-Ruzzi A, Sipos A, Bai P (2020). NRF2 in regulating cancer metabolism. Antioxid. Redox Signal..

[104] Pacher P, Schulz R, Liaudet L, Szabo C (2005). Nitrosative stress and pharmacological modulation of heart failure. Trends Pharmacol. Sci..

[105] Vida A, Abdul-Rahman O, Miko E, Brunyanszki A, Bai P (2016). Poly(ADP-ribose) polymerases in aging—Friend or Foe?. Curr. Protein Pept. Sci..

[106] Mangerich A, Herbach N, Hanf B, Fischbach A, Popp O, Moreno-Villanueva M (2010). Inflammatory and age-related pathologies in mice with ectopic expression of human PARP-1. Mech. Ageing Dev..

[107] Muiras ML, Muller M, Schachter F, Burkle A (1998). Increased poly(ADP-ribose) polymerase activity in lymphoblastoid cell lines from centenarians. J. Mol. Med..

[108] Muiras ML, Burkle A (2000). Defending genomic stability over life span: a proposed role for PARP-1. Exp. Gerontol..

[109] Berger NA, Besson VC, Boulares AH, Burkle A, Chiarugi A, Clark RS (2018). Opportunities for the repurposing of PARP inhibitors for the therapy of non-oncological diseases. Br. J. Pharmacol..

[110] Barreiro E, Gea J (2018). PARP-1 and PARP-2 activity in cancer-induced cachexia: potential therapeutic implications. Biol. Chem..

[111] Yélamos J, Moreno-Lama L, Jimeno J, Ali SO (2020). Immunomodulatory roles of PARP-1 and PARP-2: Impact on PARP-centered cancer therapies. Cancers (Basel).

[112] Bustin SA, Benes V, Garson JA, Hellemans J, Huggett J, Kubista M (2009). The MIQE guidelines: Minimum information for publication of quantitative real-time PCR experiments. Clin. Chem..

[113] Rueden CT, Schindelin J, Hiner MC, DeZonia BE, Walter AE, Arena ET (2017). Image J2: ImageJ for the next generation of scientific image data. BMC Bioinformatics.

[114] Janko L, Sari Z, Kovacs T, Kis G, Szanto M, Antal M (2020). Silencing of PARP2 blocks autophagic degradation. Cells.

[115] The Gene Ontology resource: Enriching a GOld mine. *Nucl. Acids Res*. **49**(D1), D325–D34 (2021). 10.1093/nar/gkaa1113 PMC7779012 33290552

[116] Ashburner M, Ball CA, Blake JA, Botstein D, Butler H, Cherry JM (2000). Gene ontology: Tool for the unification of biology. The Gene Ontology Consortium. Nat Genet..

[117] Eleutherio ECA, Silva Magalhães RS, de Araújo BA, Monteiro Neto JR, de Holanda PL (2021). SOD1, more than just an antioxidant. Arch. Biochem. Biophys..

[118] Davis S, Meltzer PS (2007). GEOquery: A bridge between the Gene Expression Omnibus (GEO) and BioConductor. Bioinformatics.

[119] Ritchie ME, Phipson B, Wu D, Hu Y, Law CW, Shi W (2015). limma powers differential expression analyses for RNA-sequencing and microarray studies. Nucl. Acids Res..

