# Supplementary material for: PARP2 poly(ADP-ribosyl)ates nuclear factor erythroid 2-related factor 2 (NRF2) affecting NRF2 subcellular localization
Source: Sci Rep. 2023 May 15;13:7869. doi: 10.1038/s41598-023-35076-w (PMC10185692; doi:10.1038/s41598-023-35076-w)
Supplement: Supplementary file 1 — Supplementary Information. [file 41598_2023_35076_MOESM1_ESM.pdf]

Uncropped blot images for Jankó et al. „**PARP2 poly(ADP-ribosyl)ates nuclear factor erythroid 2-related factor 2 (NRF2) affecting NRF2 subcellular localization**”

Fig. 1B

The apparent MW of PARP2 is ~62 kDa. Ame et al. JBC 274(25) 17860, 1999.

Blots were routinely cut as shown.

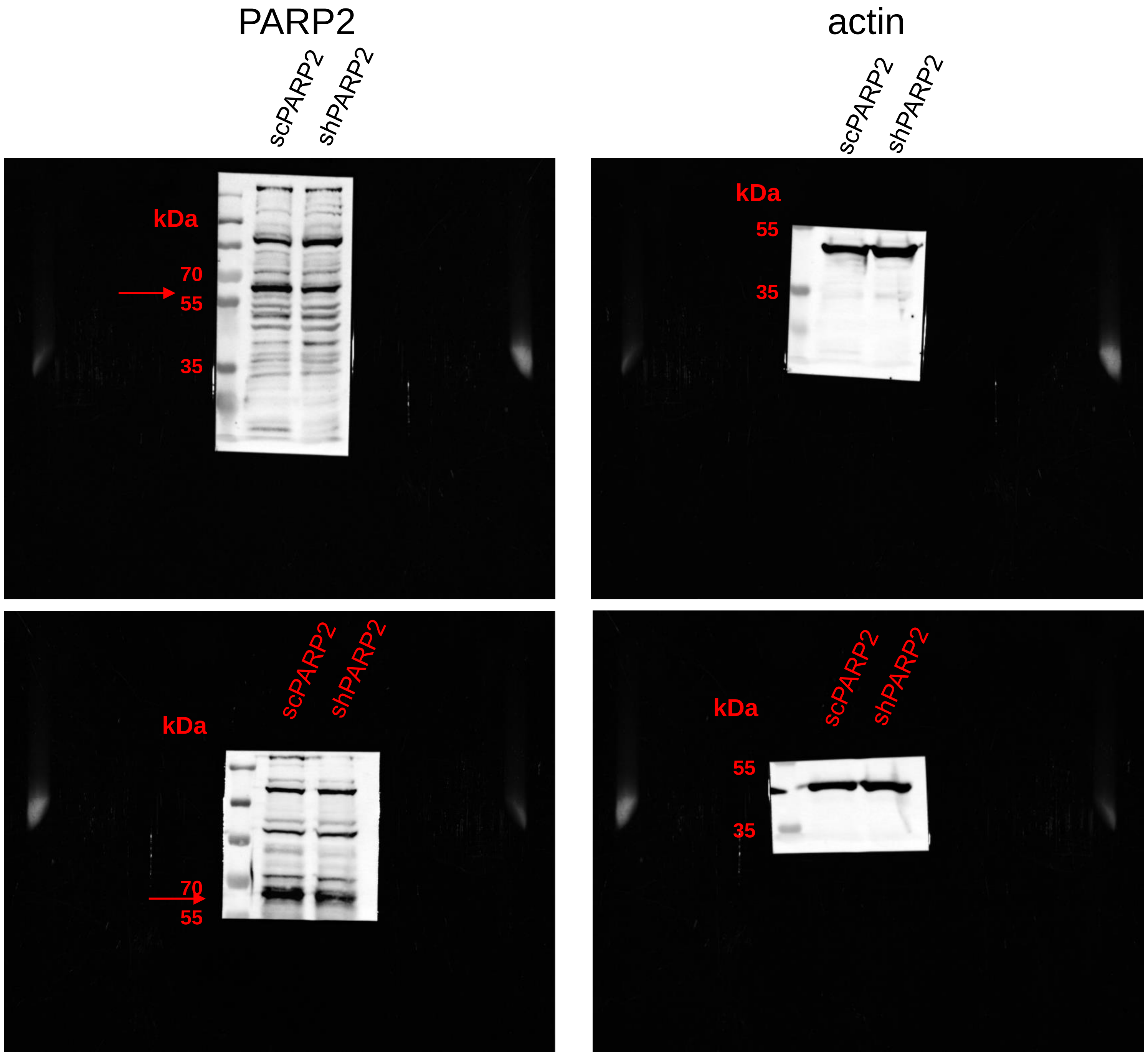

Fig. 1B

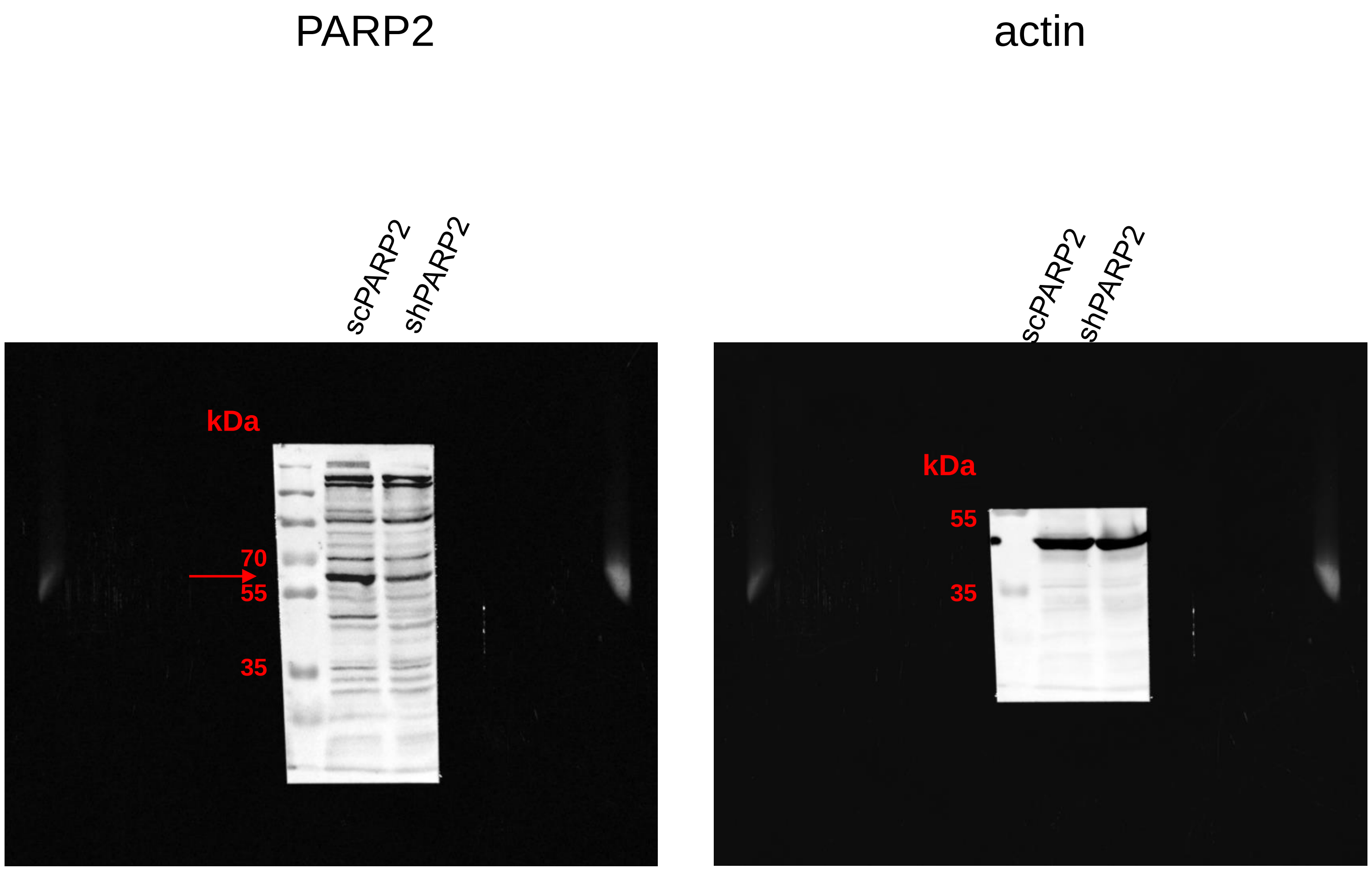
PARP2
actin
scPARP2
shPARP2
kDa
70
55
35
scPARP2
shPARP2
kDa
55
35

Nrf2 (Abcam) Western blot

Fig. 1D

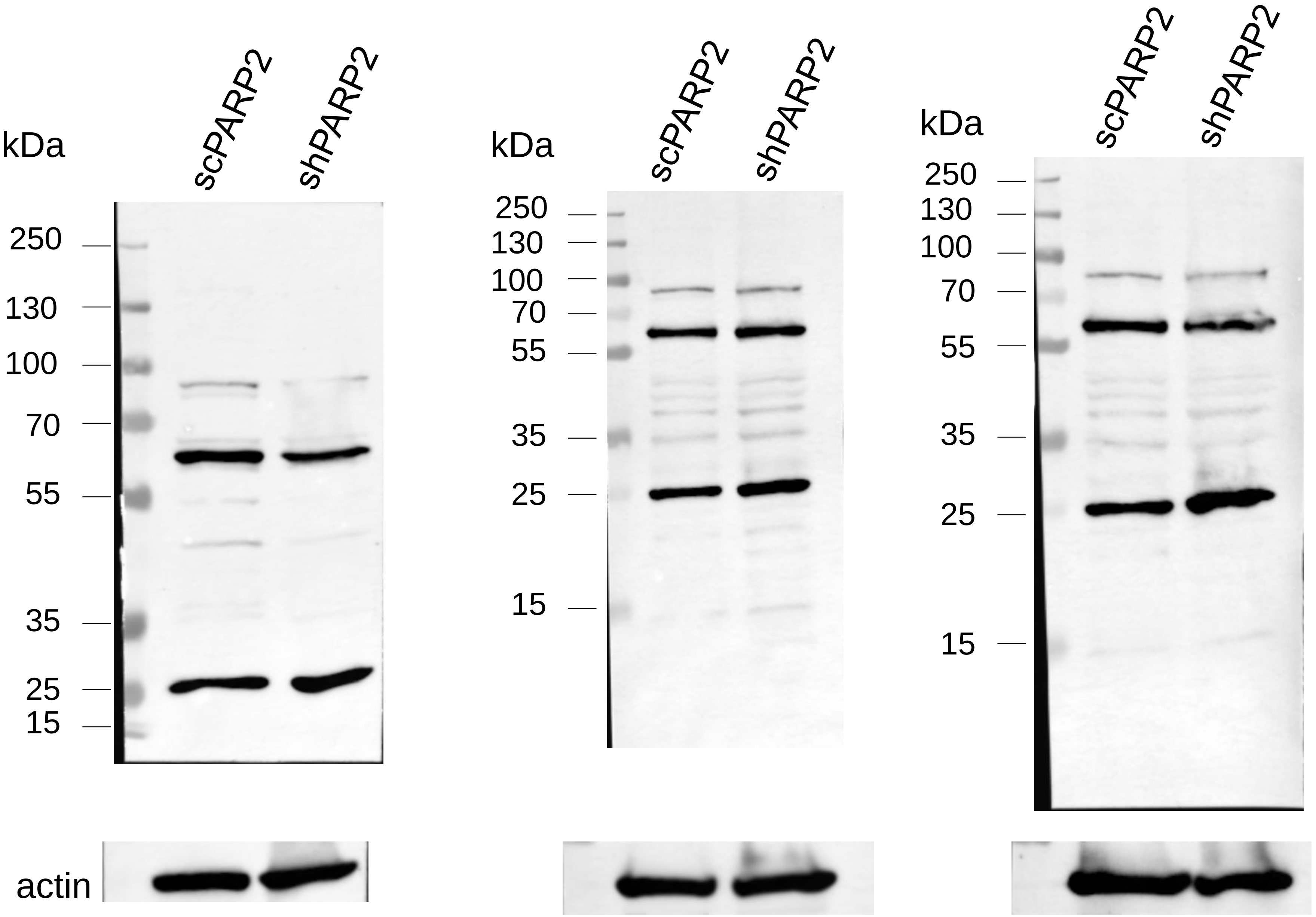

Nrf2 (Novus) Western blot

Fig. 1D

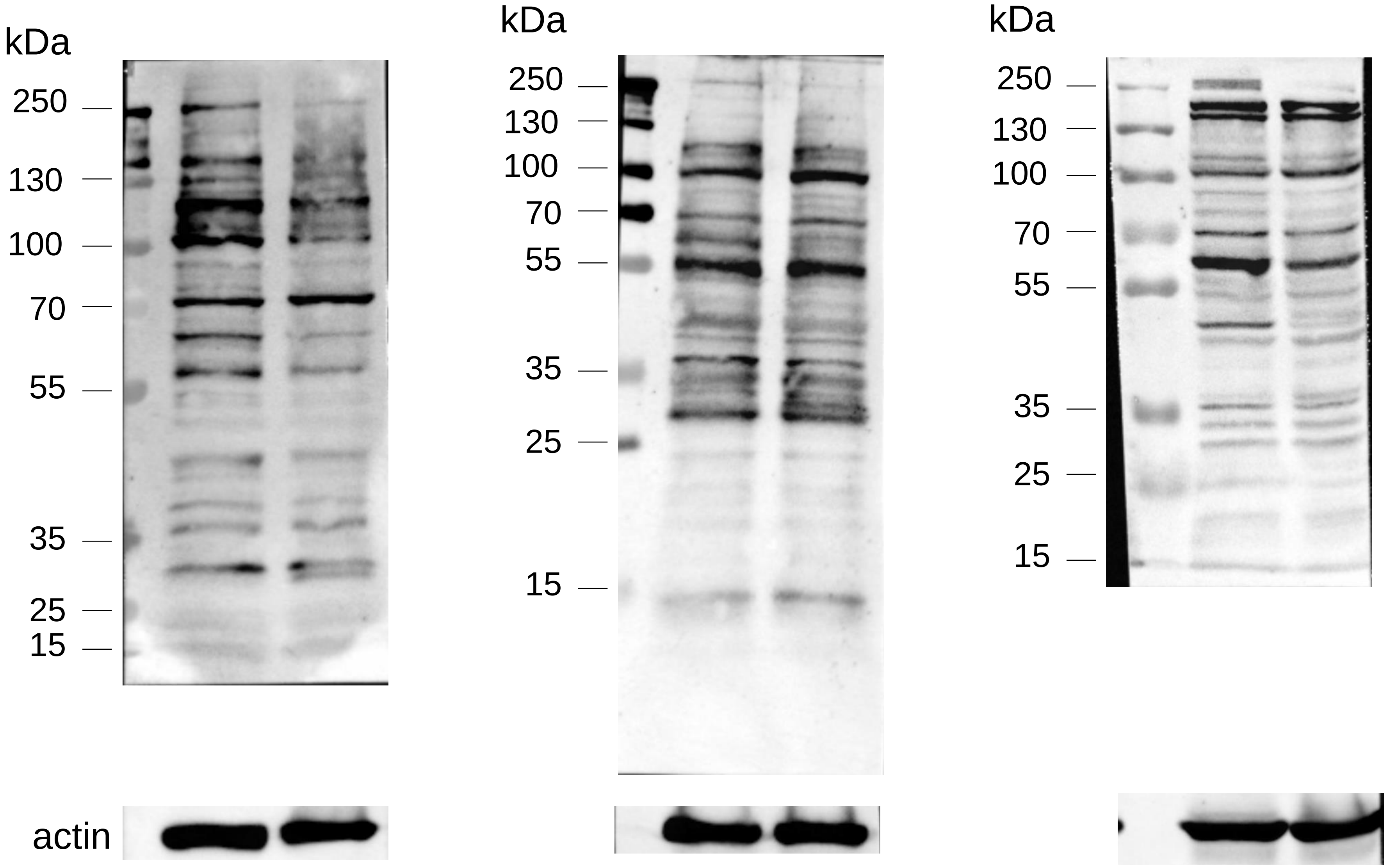

Fig. 3A

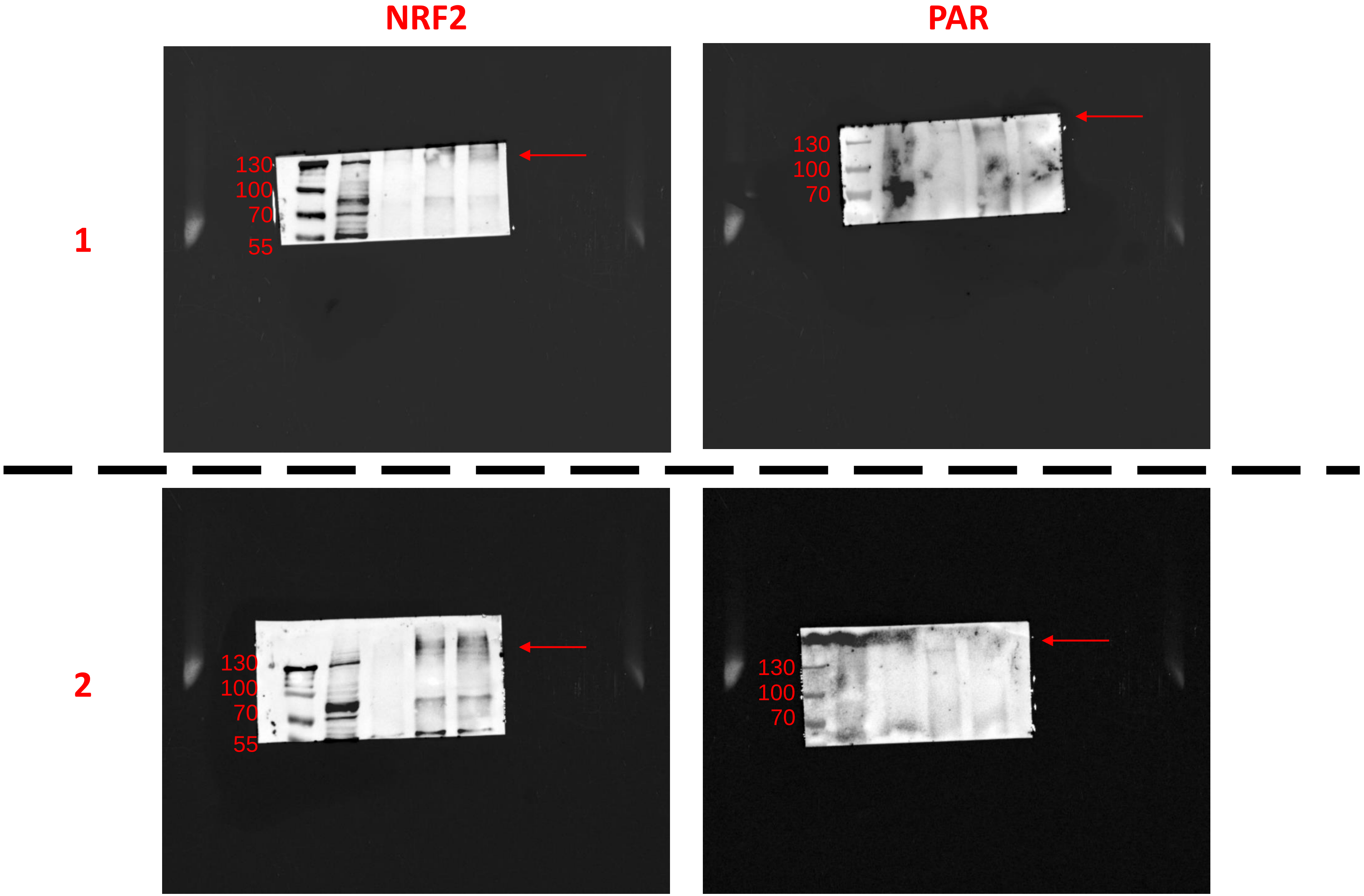

Fig. 4

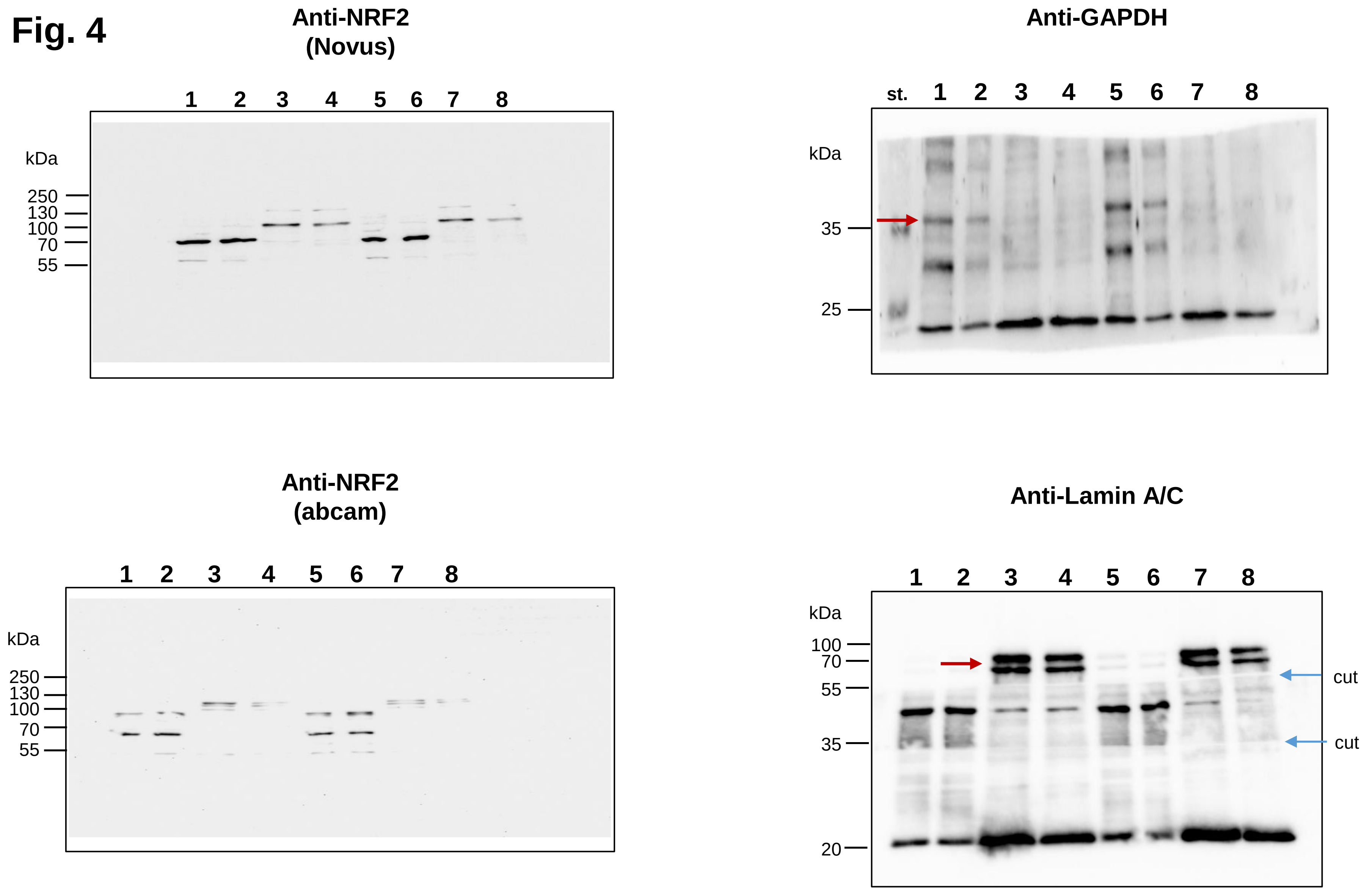

1. CTRL scPARP2 cytosol
2. CTRL shPARP2 cytosol
3. CTRL scPARP2 nucleus
4. CTRL shPARP2 nucleus
5. Olaparib scPARP2 cytosol
6. Olaparib shPARP2 cytosol
7. Olaparib scPARP2 nucleus
8. Olaparib shPARP2 nucleus
